# Do professional facial image comparison training courses work?

**DOI:** 10.1371/journal.pone.0211037

**Published:** 2019-02-13

**Authors:** Alice Towler, Richard I. Kemp, A. Mike Burton, James D. Dunn, Tanya Wayne, Reuben Moreton, David White

**Affiliations:** 1 The University of New South Wales, Sydney, Australia; 2 The University of York, York, United Kingdom; 3 The Metropolitan Police Service, London, United Kingdom; 4 The Open University, Milton Keynes, United Kingdom; Univdersity Hospital of TübingenUniversitatsklinikum Tubingen, GERMANY

## Abstract

Facial image comparison practitioners compare images of unfamiliar faces and decide whether or not they show the same person. Given the importance of these decisions for national security and criminal investigations, practitioners attend training courses to improve their face identification ability. However, these courses have not been empirically validated so it is unknown if they improve accuracy. Here, we review the content of eleven professional training courses offered to staff at national security, police, intelligence, passport issuance, immigration and border control agencies around the world. All reviewed courses include basic training in facial anatomy and prescribe facial feature (or ‘morphological’) comparison. Next, we evaluate the effectiveness of four representative courses by comparing face identification accuracy before and after training in novices (*n* = 152) and practitioners (*n* = 236). We find very strong evidence that short (1-hour and half-day) professional training courses do not improve identification accuracy, despite 93% of trainees believing their performance had improved. We find some evidence of improvement in a 3-day training course designed to introduce trainees to the unique feature-by-feature comparison strategy used by facial examiners in forensic settings. However, observed improvements are small, inconsistent across tests, and training did not produce the qualitative changes associated with examiners’ expertise. Future research should test the benefits of longer examination-focussed training courses and incorporate longitudinal approaches to track improvements caused by mentoring and deliberate practice. In the absence of evidence that training is effective, we advise agencies to explore alternative evidence-based strategies for improving the accuracy of face identification decisions.

## Background

Critical identification procedures, such as passport checks at border control and identifying offenders from CCTV surveillance footage, require facial image comparison practitioners to verify the identity of unfamiliar people by comparing faces. Errors in these situations can have serious personal and societal consequences, such as convicting an innocent person while the true perpetrator goes free. By far the most common way to mitigate these risks is for practitioners to attend training courses to improve their ability [1]. However, very little is known about this training. Official guidelines for the content of facial image comparison training courses exist (see [2, 3] and S1 Appendix), but it is unclear to what extent agencies adhere to them. More importantly, we do not know if professional training courses *improve* identification accuracy. Here, we report a comprehensive review of current professional facial image comparison training courses and report the first empirical evaluations of their effectiveness.

The only published evaluation of a professional facial image comparison training course was conducted in the 1970’s by psychologists Woodhead, Baddeley, and Simmonds [4]. Over three days, police officers were taught to take note of characteristics such as a person’s age, mannerisms and oddities; to exaggerate unique facial features in their minds so they could eliminate non-matching identities without the same features; and, to discuss and label distinctive facial features. Face identification accuracy was tested in a number of tasks before and after training using novel images. Surprisingly, the trained group did not outperform the untrained group on any of these tests. Although the effectiveness of facial image comparison training courses may have improved considerably in the decades since Woodhead et al.’s study, we know of no validation tests that have been conducted since.

This lack of empirical validation for facial image comparison training courses is particularly concerning because humans are notoriously poor at identifying unfamiliar faces. Error-rates in tests typically average 20–30% for both novices and trained practitioners [5–9]. In a live photo-to-person identity verification task for example, passport officers accepted 14% of fraudulent ID documents and rejected 6% of valid IDs [9]. In another study, passport officers searched for a target person in an array of faces selected by facial recognition software, a task they perform in their daily work to screen passport applications for identity fraud [10]. Passport officers made errors on more than 50% of decisions—the same rate as untrained novices.

In many organisations, attending a facial image comparison training course is taken as evidence of competency. The courses we evaluate here qualify practitioners within their own organisations to make critical identification decisions in police, intelligence and national security operations, at borders, and when issuing secure identity documents (e.g., passports). More generally, training is also accepted as evidence of expertise in court [11], allowing ‘trained experts’ to present highly persuasive identification evidence at trial [12]. In light of these issues, and recent calls for evidence-based practice and validation in the forensic sciences [13–16], it is critical to ask whether professional facial image comparison training courses improve identification accuracy.

## Review of current training course content

The Facial Identification Scientific Working Group (FISWG) develop best-practice guidelines for facial image comparison (see [2] and S1 Appendix). Membership comprises agencies across several countries, including the United States Federal Bureau of Investigation (FBI), Department of State, and Army; the Australian Federal Police, Passport Office, and Home Affairs; the UK Metropolitan Police Service and Home Office; and, the Netherlands Forensic Institute (NFI). FISWG’s training guidelines are available for agencies to use when developing training, and recommend that training includes: principles of comparison, automated biometric systems, image science, media, image processing, facial knowledge and legal issues.

The FISWG guidelines do not make reference to empirical research supporting the validity of the recommended training practices, so adherence to the guidelines provides no evidence that a particular training course is effective. Nonetheless, because the FISWG guidelines are internationally accepted as best-practice we used them as classification criteria to review the content of eleven professional facial image comparison training courses (see Methods for details). Nine of these courses are provided by police, intelligence, national security, passport issuance, immigration and border control agencies in Australia, Finland, the UK and USA, and two are provided by a private global biometrics company.

**Fig 1:**
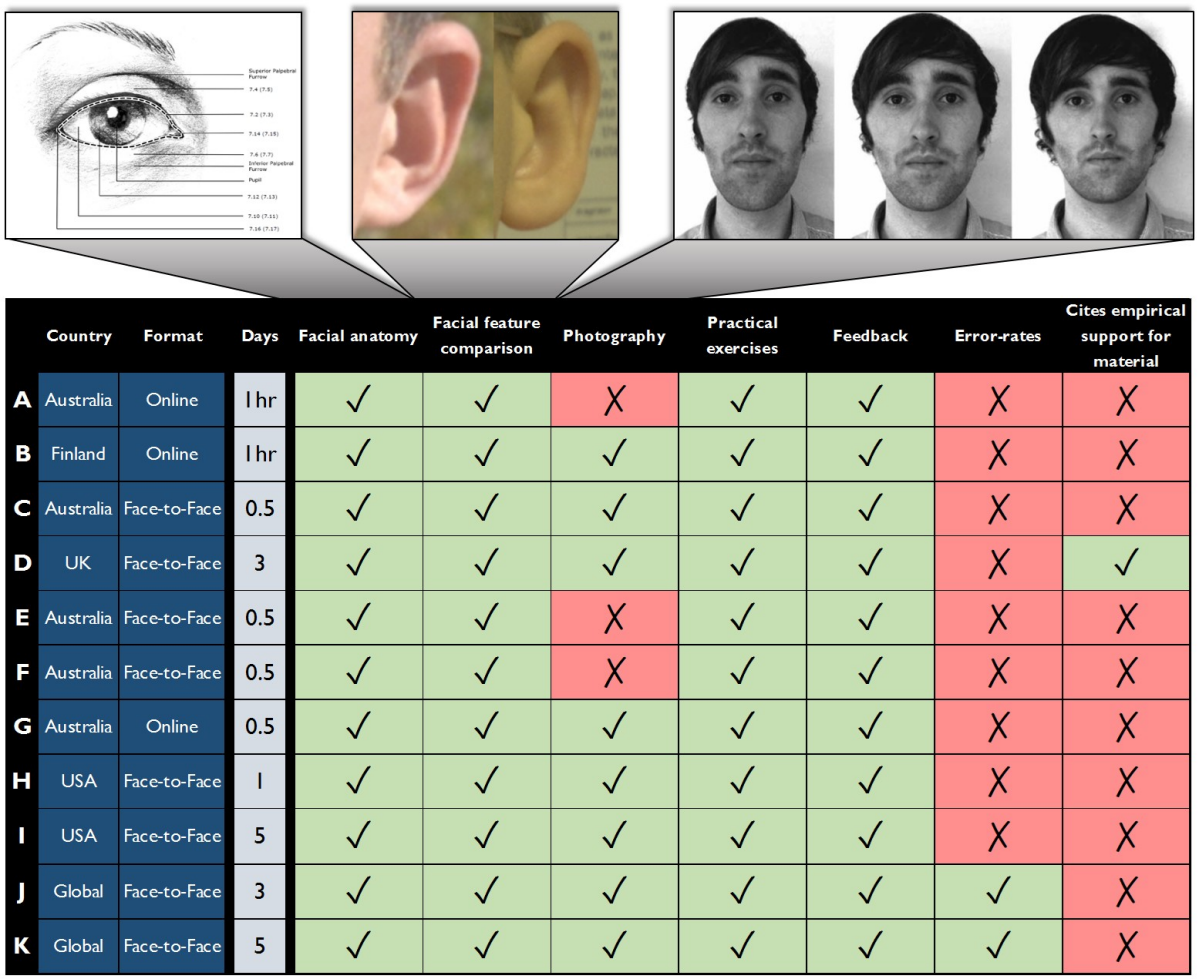
Summary of professional facial image comparison training course content. We reviewed the content of eleven professional facial image comparison training courses. All courses teach facial anatomy (e.g. top left), and encourage facial feature comparison (e.g. top centre). Most courses teach principles of photography, such as the effect of subject-to-camera distance on face photographs (e.g. top right). Notably, only two courses mention error-rates, and only one course cites empirical research to support the training material. A full version of this review table is available in supplementary materials (S2 Appendix). Illustration of the eye: Reprinted under a CC BY license with permission from Jane Wankmiller. Ear figure: Adapted from the Good, Bad and Ugly image set [18]; Photography figure: Reprinted under a CC BY license, original copyright 2013 by A. M. Burton.

A summary of training course content is provided in Fig 1. The complete content review is provided in S2 Appendix (see also [1]). We found that most training courses are presented face-to-face and delivered on a single day. All courses teach facial anatomy, describing the morphology of facial features, such as the eyes, nose, mouth, ears, face shape and facial marks (see Fig 1, top left). All courses encourage a facial feature comparison strategy or ‘morphological comparison’, whereby practitioners are directed to break the face down into its parts and compare the similarity of individual facial features (see Fig 1, top centre). Many courses also encourage a feature *classification* strategy, whereby facial features are classified according to predetermined categories (e.g., round, square, diamond face shapes), and identity judgments are based on the agreement of classifications (see [17] for an evaluation of this strategy).

Most courses describe principles of photography, noting how image capture conditions affect a person’s appearance. For example, courses describe the impact of subject factors (e.g., pose, expression, head angle) and photographic factors (e.g., camera angle, illumination, lens, zoom, camera-to-subject distance) on the resulting image (see Fig 1, top right). All courses include practical exercises and provide feedback on the accuracy of facial comparison decisions to trainees (see [19]).

It is striking that there is very little connection between these courses and behavioural studies of face identification performance. Fewer than half of the reviewed courses state that face identification is difficult despite overwhelming empirical evidence that it is. Only two courses (J and K) include reference to scientific studies of error-rates in novice and/or professional groups, and Course D is the only course to cite empirical evidence to support some of the training material [20].

## Evaluation of the effectiveness of current training courses

**Fig 2:**
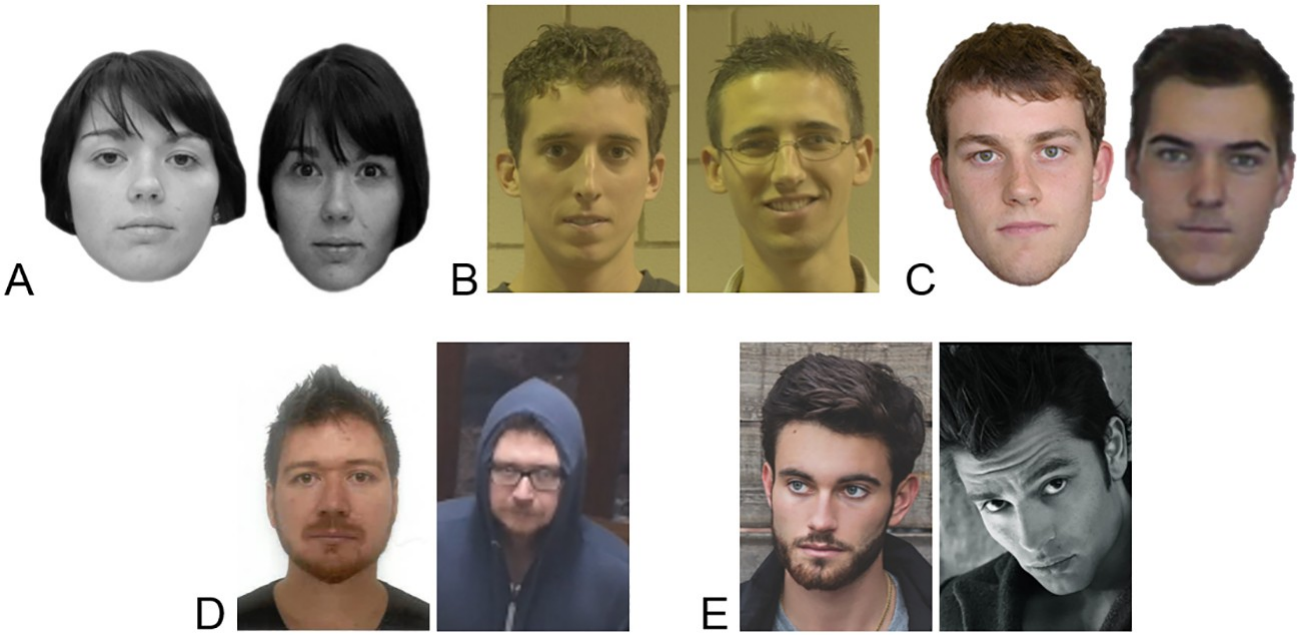
Example stimuli from the face identification tasks. Example stimuli from the (A) GFMT, (B) GBU tests and feature rating task, (C) the High-to-Low image quality test, and representative stimuli from the (D) casework test and (E) inversion test. In each test, participants were asked to decide if the photos show the same person or different people. The answers to these pairs can be found in the Acknowledgements.

We evaluated the effectiveness of four of the training courses reviewed above (Courses A, B, C & D) to determine if they improve identification accuracy. In each evaluation, we compared the training group/s to a control group of participants who received training unrelated to face identification. All evaluations employed a pre- to post-test design whereby participants completed a series of tests before and after training so that we could track changes in accuracy as a result of training. The pre- and post-tests always included the Glasgow Face Matching Test (GFMT; see Fig 2A; [6]), and other tests specifically designed to simulate the casework each training course is intended to improve. For all tests, we split items into two sets of equal difficulty using accuracy data from previous studies so that equivalent versions could be administered at pre- and post-test. In the following sections, we report only critical aspects of the design and procedure with full details provided in the Methods section.

In each training evaluation, accuracy data were analysed using ANOVA with Training (training group/s, control group) as a between-subjects factor and Test (pre-training, post-training) as a within-subjects factor. If a professional training course improves identification accuracy we would expect to observe a statistically significant interaction between Training and Test, whereby an improvement in accuracy from pre- to post-training is larger for the training group than the control group. For brevity, we only report this critical interaction and appropriate follow-up tests below. Complete analyses and complementary Bayesian analyses of null training effects are provided in S3 Appendix.

### Online training course evaluation

Courses A and B (see Fig 1) are delivered online to passport issuance and police personnel in Australia and Finland. We evaluated the effectiveness of these courses using 60 undergraduate psychology students and compared their performance to 30 control participants who completed an online workplace health and safety training course of similar duration.

All participants completed three face identification tests before and after training: (i) the Glasgow Face Matching Test (GFMT), a self-paced 20-item pairwise test of face matching ability (see Fig 2A; [6]); (ii) the GBU-unlimited test, a self-paced 30-item pairwise matching task constructed using images from the Good, Bad and Ugly (GBU) image set (see Fig 2B; [18]); and (iii) the GBU-4 seconds test where 30 image pairs from the GBU image set were presented for 4 seconds each (see Fig 2B). In each test, participants decided whether image pairs showed the same person or different people (see Methods for full details).

These three tests were chosen because they reflect the types of decisions made by practitioners in Course A and B’s host agencies. The GFMT represents optimal conditions for matching, with images taken on the same day and in similar studio-quality capture conditions, whereas the GBU tests require participants to match faces that were taken in unconstrained environmental conditions and months apart.

**Fig 3:**
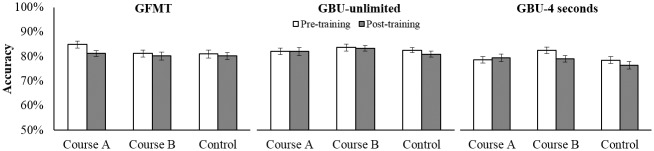
Identification accuracy before and after completing Course A or Course B. Accuracy on the GFMT, GBU-unlimited and GBU-4 seconds tests before (pre-training) and after (post-training) completing online Courses A or B, or the control training. Across all three tests there was no improvement from training. Error bars show within-subjects corrected standard error of the mean [21].

Accuracy scores on the three tests at pre- and post-training are presented in Fig 3. For all tests, the interactions between Training and Test were non-significant [GFMT: *F*<1, *ƞ*_*p*_^*2*^ = .01; GBU-u: *F*<1, *ƞ*_*p*_^*2*^ = .00; GBU-4s: *F*<1, *ƞ*_*p*_^*2*^ = .02], providing no evidence that Courses A and B improve face identification accuracy.

Complementary Bayesian analyses of the Training x Test interactions revealed the observed data are between 33 and 111 times more likely to occur when Courses A and B do not improve accuracy compared to when they do. These analyses provide between ‘very strong’ and ‘extreme’ evidence in support of the null hypothesis, i.e. that Courses A and B do not improve face identification accuracy.

It is possible that Courses A and B are not representative of facial image comparison training courses more generally. These online courses only take 1 hour to complete and are thus unable to cover topics in great detail. Further, the undergraduate students tested in this evaluation may not have been motivated to engage in the training. We address these issues in the next evaluation, by testing performance of genuine trainees before and after a half-day training course delivered face-to-face by a qualified and experienced instructor.

### Half-day face-to-face training course evaluation

Course C (see Fig 1) is designed for facial image comparison staff who verify the identity of people from high-quality front-facing photographs (e.g., passport photos). We evaluated the effectiveness of this half-day face-to-face training course using 204 genuine trainees from an Australian government agency. Participants completed the training and evaluation tests in regional offices around Australia. On average, participants had been employed in a facial image comparison role at the agency for 7 years. An additional 42 control participants recruited via Amazon’s Mechanical Turk completed a 1-hour online conflict resolution training course.

All participants completed three face identification tests before and after training: (i) the GFMT [6], as used in the previous evaluation; (ii) a High-to-Low image quality test, a 20-item pairwise matching task where high- and low-quality images were paired together (see Fig 2C); and (iii) a High-to-High image quality test, where two high-quality images were paired together. This enabled us to examine the effectiveness of training across a range of image quality conditions, not only those for which the training is designed. In each test, participants were asked to decide if two simultaneously presented photos showed the same person or different people (see Methods for full details).

**Fig 4:**
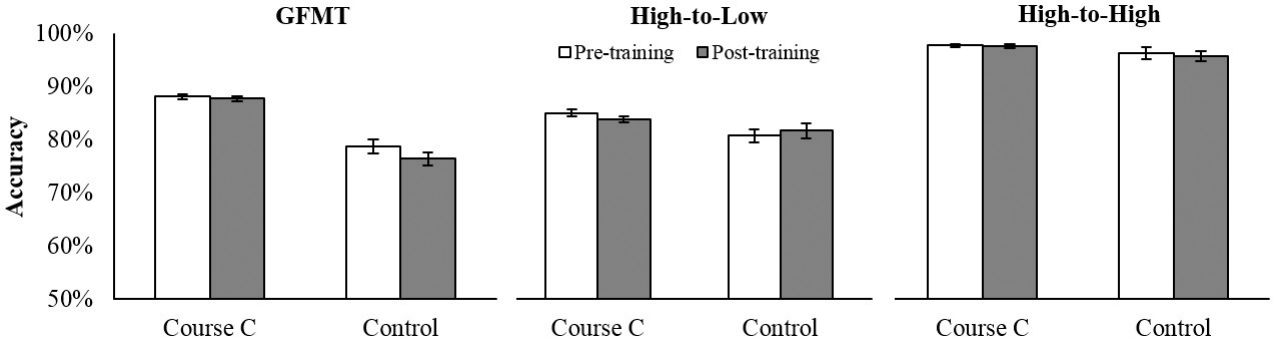
Identification accuracy of Course C trainees before and after training. Accuracy on the GFMT, High-to-Low and High-to-High image quality tests for 204 Course C trainees from an Australian government agency and 42 control participants before (pre-training) and after training (post-training). Across all three tests there was no improvement from training. Error bars show within-subjects corrected standard error of the mean [21].

Accuracy scores on the three face identification tests at pre- and post-training are presented in Fig 4. Across all three tests, the interactions between Training and Test were non-significant [GFMT: *F*(1, 244) = 1.48, *p* > .05, *ƞ*_*p*_^*2*^ = .01; High-to-Low: *F*<1, *ƞ*_*p*_^*2*^ = .00; High-to-High: *F*<1, *ƞ*_*p*_^*2*^ = .00]. We therefore found no evidence that Course C improves identification accuracy.

Bayesian analyses of the Training x Test interactions revealed the observed data are between 5 and 14 times more likely to occur when Course C does not improve accuracy compared to when it does. These analyses provide between ‘moderate’ and ‘very strong’ evidence in support of the null hypothesis, i.e. that Course C does not improve face identification accuracy.

More than half of Course C trainees reported having previously received training in facial image comparison. To determine if previous training diluted the interaction effect we repeated the analyses above including only those who had not received prior training (*n* = 99), but the interactions between Training and Test for these participants were also non-significant [GFMT: *F*<1, *ƞ*_*p*_^*2*^ = .00; High-to-Low: *F*(1, 139) = 2.17, *p* > .05, *ƞ*_*p*_^*2*^ = .02; High-to-High: *F*<1, *ƞ*_*p*_^*2*^ = .00].

Bayesian analyses of the Training x Test interactions for previously untrained participants revealed the observed data are between 4 and 13 times more likely to occur when Course C does not improve accuracy compared to when it does. These analyses provide further evidence that Course C does not improve face identification accuracy.

After participants completed their training course and before the post-training face identification tests, we asked them whether they believed training had improved their identification accuracy. Interestingly, despite neither course improving accuracy, 93% of Course C trainees believed they had improved as a result of training, compared to 31% of the control group. This suggests that people do not have insight into the effectiveness of facial image comparison training, and that perceived benefit of training is unrelated to its actual benefit.

Although there is very strong evidence that this half-day course does not improve face identification accuracy, it is possible that longer and more intensive courses are more effective. Our next evaluation therefore tested the effectiveness of Course D, an interactive 3-day course which focusses on the unique feature-by-feature comparison strategy used by facial examiners in forensic settings.

### 3-day face-to-face training course evaluation

Course D (see Fig 1) is tailored to police officers and civilian staff from a UK police service who identify people using both high-quality controlled images (e.g., mugshots) and low-quality unconstrained images (e.g., CCTV footage) in criminal investigations and intelligence operations. This course provides an introduction to the unique feature-by-feature comparison strategy used by facial *examiners* in forensic settings. Facial examiners are a specialist group of facial image comparison practitioners who conduct slow, systematic, feature-based comparisons, often as part of criminal investigations (see [20, 22, 23]). Course D therefore places much greater emphasis on the systematic comparison of facial features than previous courses.

We evaluated the effectiveness of this 3-day face-to-face training course using 32 genuine Course D trainees from a UK police service. Of the 72% who already worked in facial image comparison roles, average time on the job was 1 year. We compared their performance to 20 control participants who completed Course B (see Fig 1), shown in the online training course evaluation to be ineffective, and the face shape classification portion of Course H (see Fig 1), which is also ineffective (see [17]).

All participants completed two face identification tests before and after training: (i) a modified version of the GFMT [6] (see Fig 2A and S3 Appendix); and (ii) a casework test, a 24-item pairwise matching test consisting of a high-quality front-facing image (e.g. mugshot) and a poorer quality front-facing image (e.g. CCTV or pixelated), some of which were taken several years apart (see Fig 2D). The casework test is representative of forensic facial examination casework and was supplied by Course D’s host agency (see Methods for details). Course D trainees had not previously seen the images in the casework test.

Recent studies show that facial examiners are more accurate than novices on face identification tasks [20, 22–24]. Importantly, these experts also show *qualitative* differences in their performance: extracting more diagnostic identity information from facial features than novices [20], and showing smaller face inversion effects [20, 22] (see S3 Appendix for details of the inversion effect). These qualitative differences appear to reflect a greater reliance on feature-level details when comparing face images.

In light of these findings, and Course D’s focus on the feature comparison strategy used by facial examiners, we also tested whether Course D leads to qualitative changes in performance by including two tests that measured the extent to which participants engaged in feature analysis. First, we administered a *feature rating task* described by Towler, White and Kemp [20]. This is a 60-item pairwise matching test where participants rate the similarity of facial features on a 5-point scale from 1 (*very dissimilar appearance*) to 5 (*very similar appearance*). Second, we included an *inversion test* to measure the face inversion effect using a 24-item pairwise matching test consisting of images of male models presented upright or inverted [25] (see Methods for full details).

**Fig 5:**
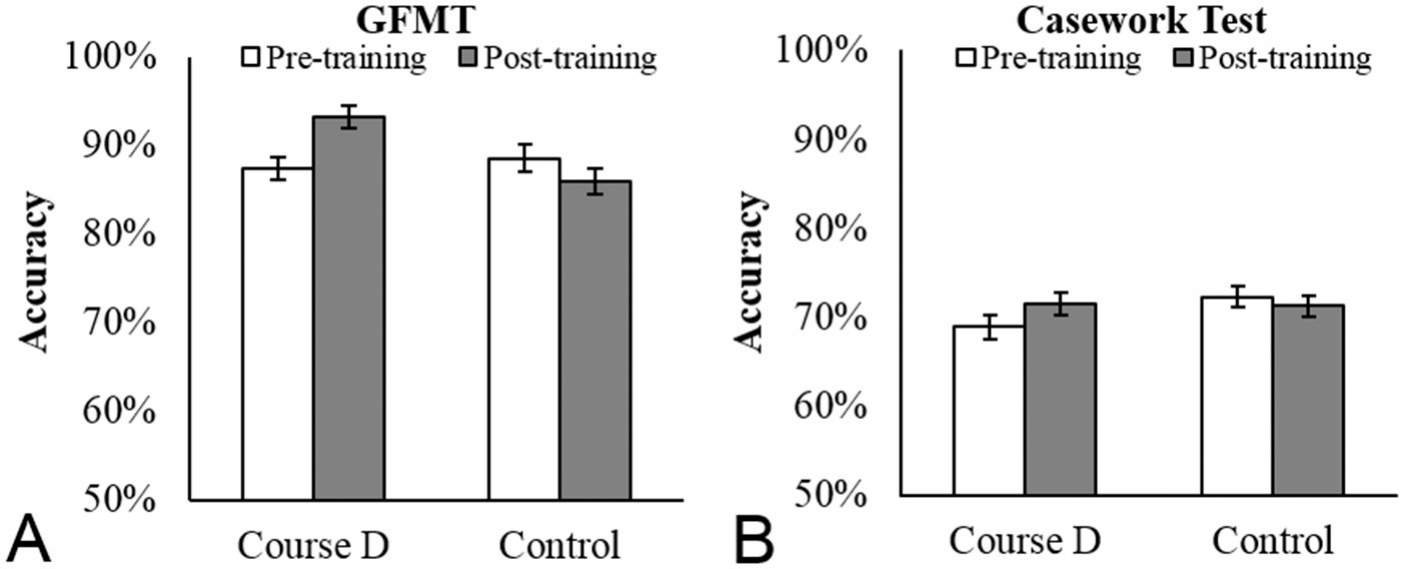
Identification accuracy of Course D trainees before and after training. Accuracy on the GFMT (A) and casework test (B) at pre- and post-training for 32 Course D trainees from a UK police service and 20 control participants. Course D trainees showed significant improvement on the GFMT but not the casework test. Error bars show within-subjects corrected standard error of the mean [21].

*Glasgow Face Matching Test*. Accuracy data on the GFMT are presented in Fig 5A. The interaction between Training and Test was significant [*F*(1, 47) = 4.69, *p* < .05, *ƞ*_*p*_^2^ = .09]. Simple main effects analyses confirmed a significant benefit of training for Course D trainees [*F*(1, 47) = 5.47, *p* < .05] but not for the control group [*F*<1]. Course D trainees’ performance on the GFMT post-training was however still far from perfect (93%), despite optimal conditions for matching (see [6]).

*Casework Test*. Accuracy data on the casework test are presented in Fig 5B. The interaction between Training and Test was non-significant [*F*<1, *ƞ*_*p*_^2^ = .02], indicating that Course D did not improve identification accuracy on comparisons representative of forensic casework. Performance on the casework test was also notably low, with Course D trainees achieving just 72% accuracy after training—comparable to novice control participants who achieved 71%.

Bayesian analysis of the Training x Test interaction revealed the observed data are 12 times more likely to occur when Course D does not improve accuracy compared to when it does, providing ‘strong’ evidence in support of the null hypothesis, i.e. that Course D does not improve face identification accuracy on the casework test.

**Fig 6:**
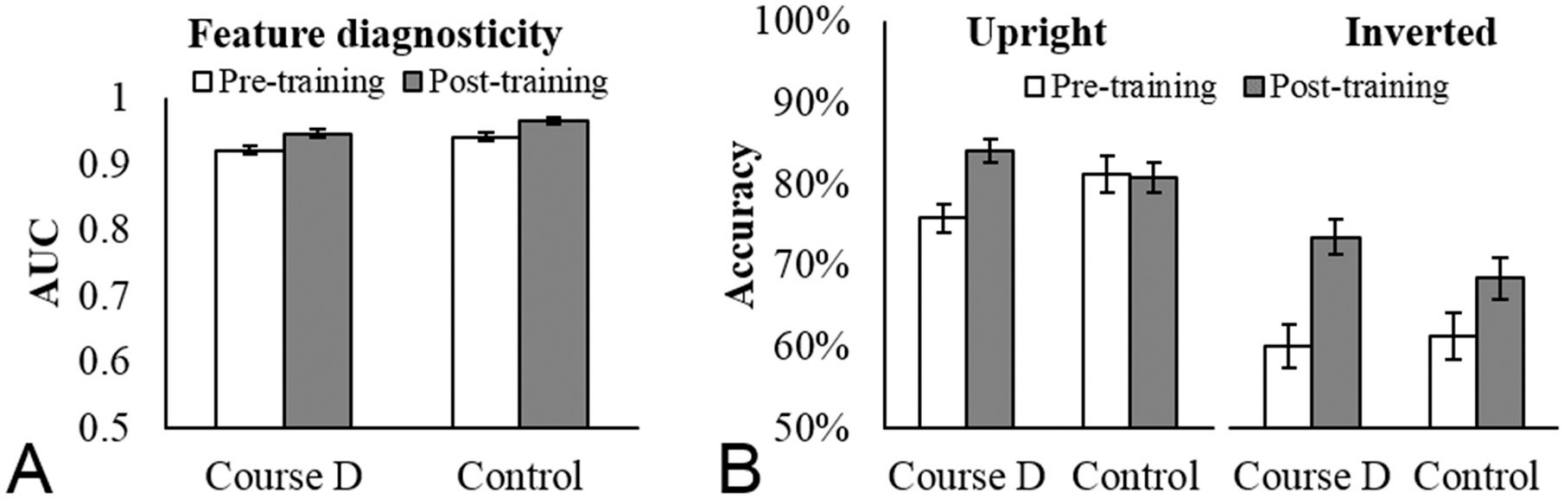
Qualitative measures of facial image comparison expertise before and after training. (A) The extent to which facial feature similarity ratings predict same/different identities (AUC) for Course D trainees from a UK police service and control participants at pre- and post-training. (B) Accuracy on the inversion test at pre- and post-training for Course D trainees and control participants. Course D trainees and control participants show equivalent feature diagnosticity and inversion effects, indicating that Course D did not produce the qualitative indicators of facial image comparison expertise. Error bars show within-subjects corrected standard error of the mean [21].

*Feature Rating Task*. Following Towler, White and Kemp [20], we calculated the extent to which participants’ feature similarity ratings on the feature rating task were diagnostic of identity by measuring Area Under the ROC Curve (AUC; see Fig 6A). The interaction between Training and Test was non-significant [*F*<1, *ƞ*_*p*_^2^ = .00], indicating that feature similarity ratings were equally diagnostic of identity for the Course D trainees as the control participants. Course D therefore did not improve trainees’ ability to extract diagnostic identity information from facial features.

*Inversion Test*. We analysed accuracy on the inversion test using an ANOVA with Orientation (upright, inverted) as an additional within-subjects factor (see Fig 6B). The interaction between Orientation, Training and Test was non-significant [*F*<1, *ƞ*_*p*_^2^ = .00], indicating equivalent inversion effects for Course D trainees and control participants.

Although not initially a comparison of interest, we found that the interaction between Training and Test on the inversion test was significant [*F*(1, 48) = 4.11, *p* < .05, *ƞ*_*p*_^2^ = .08] (see Fig 6B). Simple main effects analyses revealed a significant improvement pre- to post-training for Course D trainees [*F*(1, 48) = 21.42, *p* < .001] but not for the control participants [*F*(1, 48) = 1.35, *p* > .05].

Unlike the two previous evaluations, we found some evidence of training effects in this 3-day face-to-face training course evaluation. Course D trainees showed significant improvements on a modified version of the GFMT and the inversion test. However, we did not observe improvement on the casework test, despite this test being representative of the types of decisions Course D is designed to improve. In addition, Course D did not produce the qualitative changes associated with expertise in facial image comparison: increased diagnosticity of facial feature similarity ratings and a reduced inversion effect [20, 22].

## General discussion

This paper provides the first comprehensive evaluation of the effectiveness of professional facial image comparison training courses. In a rigorous review of training courses used by the international community, we found that training typically includes facial anatomy, feature comparison and photography (see Fig 1 and S2 Appendix). The courses evaluated here were representative of accepted practice in facial image comparison training, and so results are likely to be indicative of the effectiveness of professional training courses more generally.

We found overwhelming evidence that short training courses (Courses A, B and C) do not improve face identification accuracy. This is despite short courses being used routinely by government agencies around the world and complying with FISWG training guidelines (see S1 Appendix). Of the six pre- to post-training comparisons in the online and half-day training course evaluations, involving both novices (*n* = 132) and genuine trainees (*n* = 204), we found no significant improvements in accuracy after training and very strong support for the null hypothesis that short training courses do not improve face identification accuracy. A priority for future research is therefore to identify evidence-based training methods that can improve the effectiveness of short professional training courses.

We did find some evidence of accuracy improvement in Course D. This was a 3-day training course designed to introduce trainees to the forensic feature-by-feature comparison strategy used by facial examiners (see [20, 22, 23]). Genuine trainees (*n* = 32) showed small but significant improvements in accuracy on a modified version of the GFMT and the inversion test, but not on the casework test. Given that Course D focussed heavily on the analysis of feature-level details, it is possible that improvement was not detected on the casework test because the images were lower quality than those in the GFMT and inversion test—low quality images may not contain sufficient detail for fine-scale analysis to be beneficial. Nevertheless, it is concerning that Course D did not improve accuracy on the task that most closely approximated the task trainees are required to perform in daily work.

The accuracy improvements observed in Course D are consistent with previous work showing that feature-by-feature comparison improves face identification accuracy in novices [20]. It is also consistent with the fact that facial examiners—who receive extensive training in feature comparison—tend to outperform novices (e.g., [20, 22–24]). However, the superior accuracy experienced by facial examiners is typically accompanied by qualitative differences in their performance. In previous work, examiners’ ratings of facial feature similarity were more diagnostic of identity [20] and their accuracy was less impaired when face images were presented upside down compared to novices [20, 22]. We found no evidence that Course D produced changes in these qualitative measures of facial image comparison expertise.

The improvements observed in Course D were small, inconsistent across tests and not accompanied by qualitative markers of expertise in facial examination. As a result, further research is necessary to explore the development of face identification expertise and to understand the basis of examiners’ superior accuracy. In light of our results, it is likely that the superior accuracy of facial examiners reported in previous work may be due to some factor other than the formal training courses they attend. For example, examiners may be more naturally talented in face identification, having either been selected for the role or having nominated themselves for the role due to a perceived talent for, or interest in, the task. Similarly, higher levels of motivation may also contribute to their superior accuracy [26]. However, these possibilities do not account for the qualitative differences observed in their performance in previous work [20, 22].

An alternative possibility is that facial examiners receive additional sources of training that were not captured by the training courses evaluated in our study. First, the training received by facial examiners is more rigorous than that provided by Course D. Second, facial examiners’ training also includes extensive on-the-job mentoring from more senior analysts, which may be critical in developing their expertise. Supporting this, research by Dowsett and Burton [25] shows that working collaboratively on facial comparison decisions with high-performing individuals improves the accuracy of low-performers in subsequent tests. Similarly, mentors provide examiners with feedback on the accuracy of their decisions and feature-based analysis, which may also facilitate learning [19].

Our approach to evaluating the effectiveness of training courses was not designed to capture these sources of learning. However, previous studies have examined the relationship between the length of time employed in a professional role and face identification accuracy to understand the role of on-the-job learning [9, 10, 27, 28]. These studies all show no improvement with more professional experience, suggesting that on-the-job learning in these particular roles does not help develop expertise. However, none of these studies included facial examiners. Instead, participants in these studies were facial *reviewers*–non-specialist facial image comparison practitioners who perform fast, high-volume face identifications (e.g. at border control). Facial reviewers do not typically have the same opportunities for mentoring, feedback and extensive practice as facial examiners, and tend to rely completely on training courses like those reviewed here. Differences in the availability of ongoing on-the-job training and opportunities for deliberate practice (see [29]) may explain why facial examiners tend to outperform facial reviewers [10, 23]. The development of expertise in facial examination therefore remains an important question for future research, and longitudinal approaches would enable greater clarity on whether expertise in this task can develop over time (see [30]).

In light of our results, it will be important for organisations that offer facial image comparison training courses to evaluate the effectiveness of their courses, and to incorporate evidence-based training practices, such as mentoring and feedback, where possible (see [19, 25]). This is especially important because our results show that 93% of Course C trainees believed they had improved as a result of training, despite this training not improving accuracy. In general, people have very little insight into their own face identification ability [31–34], and tend to believe they are better at the task than they really are [35]. Given that so few of the training courses reviewed here made reference to the difficulty of unfamiliar face identification (e.g., [9, 10]), ineffective training could strengthen people’s misplaced confidence in their ability to accurately judge the identity of unfamiliar faces.

Finally, organisations should consider alternative approaches to improving face identification accuracy in the workplace (see [36]). Recruiting people with natural aptitude in face identification tasks [37, 38], statistical aggregation of judgments made by multiple people [39, 40], and combinations of humans and face recognition algorithms [23] have all been shown to produce substantial boosts to accuracy.

## Methods

### Review of current training course content

#### Courses

We reviewed training materials from eleven facial reviewer training courses that are currently in use or were recently used by government agencies in Australia, Finland, the UK and USA, and a private global biometrics company. These training materials consisted of presentation slides or interactive online training modules. We did not have access to course material that might ordinarily be delivered verbally by an instructor.

#### Classification criteria

FISWG’s training guidelines for facial examiners include all recommendations for facial reviewers (see [2] and S1 Appendix). For completeness, we therefore classified each training course according to the facial examiner guidelines. Each recommendation in the guidelines was used as a criterion for the review, resulting in 69 initial criteria. An additional 17 criteria were added to cover content which did not fall into one of the existing criteria, or in response to the need for evidence-based training practices. Each training course was therefore classified according to a total of 86 criteria (see S2 Appendix for the criteria).

#### Classification method

Training courses were classified as having met each criterion either ‘not at all’, ‘briefly’ or ‘in detail’ by two raters. Disagreement between the raters was resolved by joint review of the material. Classification of ‘not at all’ indicated that there was no mention of the criteria. ‘Briefly’ indicated that the criterion was mentioned but that no additional description or explanation was provided. Classification of ‘in detail’ indicated that the criterion was described in some depth. To illustrate the difference, below is a representative example of a ‘briefly’ and ‘in detail’ classification, respectively, for the criterion regarding the effect of aging on facial appearance.

Briefly: *“Aging will change the appearance of the face*.*”*

In detail: *“As people age*, *their skin loses elasticity*. *Cheeks appear more hollow*, *noses and earlobes droop and appear larger*. *Skin sags around the jawline*, *hairline recedes and wrinkles become more pronounced*.*”*

### Online training course evaluation

#### Participants

Ninety Australian undergraduate psychology students (Mean age = 19 years, *SD* = 3 years) participated in return for course credit. They were randomly allocated to one of three conditions, such that 30 participants were in each. In two conditions, participants completed Course A or Course B (see Fig 1). In a control condition, participants completed an online workplace health and safety course.

#### Materials & procedure

All participants completed three tests of face matching ability before and after completing the training: the GFMT (20 trials), GBU-unlimited (30 trials) and GBU-4 seconds (30 trials). The GFMT was always completed first. The order of the GBU-unlimited and GBU-4 seconds was counterbalanced across participants, but held constant pre- to post-training.

The Glasgow Face Matching Test (GFMT) is a standardised psychometric test of face matching ability [6]. The short version consists of 40 cropped greyscale face pairs captured on the same day under controlled studio lighting conditions (see Fig 2A). Following Towler, White and Kemp [17], the GFMT was split into two equally difficult 20-item versions (10 match, 10 non-match). Allocation of each version to the pre- and post-training was counterbalanced across participants. Participants were simultaneously presented with face pairs and asked to decide if they showed the same person or different people.

The GBU tests were created using images from the Good, Bad and Ugly image set [18], and contained a subset of the images from the Expertise in Facial Comparison Test (EFCT; see [22]). These images are captured in frontal pose on different days, and are free to vary in regards to expression, hairstyle, lighting conditions, and location (see Fig 2B). We created four equally difficult 30-item versions (15 match, 15 non-match) using human performance data from O’Toole, An, Dunlop, and Natu [41]. Allocation of each version to the pre- or post-training and GBU-unlimited and GBU-4 seconds was counterbalanced across participants. In the GBU tests participants were simultaneously presented with face pairs and asked to decide if they showed the same person or different people. In the GBU-unlimited, the image pairs remained onscreen until participants made a response. In the GBU-4 seconds, the image pairs were only visible for 4 seconds but participants could respond before or after the images were removed.

### Half-day face-to-face training course evaluation

#### Participants

Participants were 204 facial image comparison practitioners employed by Course C’s Australian government host agency (Mean age = 44 years, *SD* = 11 years). Participants completed the training and evaluation tests in regional offices around Australia. Participants had been employed in a facial image comparison role at the agency for an average of 7 years (range: 0 to 35 years). One hundred and five trainees (51%) reported they had previously completed facial image comparison training. Course C trainees were paid their normal wage.

Forty-two control participants recruited via Amazon’s Mechanical Turk completed an online conflict resolution training course and were paid US$2 for their time (Mean age = 38 years, *SD* = 12 years). None reported having previously completed any face recognition related training.

#### Materials & procedure

Participants completed three face matching tests before and after training: the GFMT (20 trials), the High-to-Low image quality test (20 trials), and the High-to-High image quality test (20 trials). Test order was randomised for each participant.

To create the High-to-Low and High-to-High image quality tests we took a high resolution digital SLR photograph of 80 target identities wearing a neutral expression. In the High-to-Low test, the target photo was paired with a low resolution front-facing digital video still taken on the same day (see Fig 2C). In the High-to-High image quality test, the target photo was paired with a high resolution digital video still taken on the same day. All images were digitally edited to remove the background and any face paraphernalia (e.g., earrings). We created two equally difficult 20-item versions (10 match, 10 non-match) of the High-to-High and High-to-Low image quality tests. Allocation of each version to pre- and post-training was counterbalanced across participants.

Participants completed a short questionnaire about their employment history, previous training and demographics, and then completed the pre-training tests. In all tests, participants saw two simultaneously presented face photographs for 30 seconds and decided if the photos showed the same person or two different people. Participants could respond before or after the images were removed. Participants then completed their training, a questionnaire about the training, and the post-training tests.

### 3-day face-to-face training course evaluation

#### Participants

Participants were 32 police officers and civilian staff employed by Course D’s UK police service host agency (Mean age = 40 years old, *SD* = 9 years). Twenty-three (72%) trainees reported making facial image comparison decisions as part of their job, and 29 (91%) reported they would do so after completing the training. For those already employed in a facial image comparison role, average time on the job was 1 year (range: 0 to 7 years). Four (13%) trainees reported they had previously received training in facial image comparison. Course D trainees were paid their normal wage. Some trainees’ data files were corrupt and could not be analysed. Exact sample sizes for each test are outlined in S3 Appendix.

Control participants were 20 British undergraduate psychology students (Mean age = 21 years, *SD* = 4 years) who were paid £30 for their time. In an effort to equate motivation between the groups, the highest-performing control participant across the pre- and post-training tests received an additional £50 Amazon voucher. Participants were only told about this incentive after they had registered for the study. No control participants reported having previously received training in facial image comparison.

#### Materials & procedure

Training and testing of Course D trainees spanned 4 days. On Day 1, trainees completed pre-training tests and began the training. Training ran on Days 1, 2 and 3. On Day 4, participants completed the post-training tests. To equate the groups’ experience as much as possible, control participants completed the pre-training tests on Day 1 and mock training on Days 2 and 3. Mock training on Day 2 consisted of Course B (see Fig 1), shown in the online training course evaluation to be ineffective. Mock training on Day 3 consisted of the face shape classification portion of Course H (see Fig 1), which we have previously shown to be ineffective (see [17]). Control participants completed the post-training tests on Day 4.

All participants completed four tasks before and after training. To test for quantitative changes in face identification ability, participants completed the modified GFMT (17–18 trials) and the casework test (24 trials). To test for qualitative changes in feature processing, participants also completed the feature rating task (60 trials) and an inversion test (36 trials). Task order was randomised for each participant, but held constant pre- to post-training. The only exception was the casework test (see below). Participants also completed a modified version of the Cambridge Face Memory Test, embedded figures test, matching familiar figures test, and the Navon task. Full details of these tasks are available in S3 Appendix.

*Glasgow Face Matching Test*. The two equally difficult halves of the GFMT used in the previous evaluations were adapted to exclude identities used in the Cambridge Face Memory Test—Australian (see [42]). One version of this modified GFMT contained 17-items (9 match, 8 non-match) and the other contained 18-items (10 match, 8 non-match). Test version order was randomly allocated to pre- and post-training for each participant. On each trial, participants decided if two simultaneously presented photos showed the same person or two different people.

*Casework Test*. The casework test consisted of a high-quality image (e.g. mugshot) and a poorer quality image (e.g. CCTV or pixelated), provided by Course D’s host agency. The test had not been previously seen by trainees. We created two equally difficult versions of the test, consisting of 24 trials each (12 match, 12 non-match). One version of the casework test was always administered at pre-training, and always as the last test, as it formed the basis of a pre-existing feedback training module. On each trial, participants decided if the photos showed the same person or two different people.

*Feature Rating Task*. The feature rating task (see [20] for full details) consists of challenging high-quality face pairs from the GBU image set [18] presented in two blocks of trials: no ratings (30 trials) and ratings (30 trials). In the no ratings block, participants decide if two simultaneously presented photos show the same person or different people. In the ratings block, participants rate the similarity of 11 facial features (e.g., ears, eyes, nose etc.) on a 5-point scale from 1 (*very dissimilar appearance*) to 5 (*very similar appearance*) before making a same/different identity decision.

*Inversion Test*. The inversion test consists of pairs of high-quality frontal face photographs of male models (see [25] for full details) presented in two blocks. In the upright block face pairs were presented upright, and in the inverted block face pairs were presented upside down. We removed 10 image pairs from the model image set that were used in a pre-existing facial feature analysis exercise during training (see S3 Appendix for details of this exercise). We then created four equally difficult versions, each containing 12 trials (6 match, 6 non-match). Two versions were randomly assigned to the upright and inverted blocks at pre- and post-training for each participant. On each trial, participants decided if the photos showed the same person or two different people. Details about the inversion effect can be found in S3 Appendix.

### Ethics statement

This research was approved by the Human Research Ethics Committee at the University of New South Wales and the Departmental Ethics Committee at the University of York. The individuals pictured in this manuscript have been sourced from publically available tests, have provided written informed consent for their image to be used, or the images were downloaded from the internet and marked as available for reuse.

## Supporting information

S1 Appendix FISWG training guidelines (v1.1 2010.11.18). (PDF)

S2 Appendix Training course content review table. (XLSX)

S3 Appendix Supplementary analyses and task details. (DOCX)

S1 Dataset Training course evaluation data. (XLSX)
