# Supplementary material for: Do professional facial image comparison training courses work?
Source: PLoS One. 2019 Feb 13;14(2):e0211037. doi: 10.1371/journal.pone.0211037 (PMC6373902; doi:10.1371/journal.pone.0211037)
Supplement: S1 Appendix — (PDF) [file pone.0211037.s001.pdf]

Disclaimer:

As a condition to the use of this document and the information contained herein, the Facial Identification Scientific Working Group (FISWG) requests notification by e-mail before or contemporaneously to the introduction of this document, or any portion thereof, as a marked exhibit offered for or moved into evidence in any judicial, administrative, legislative, or adjudicatory hearing or other proceeding (including discovery proceedings) in the United States or any foreign country. Such notification shall include: 1) the formal name of the proceeding, including docket number or similar identifier; 2) the name and location of the body conducting the hearing or proceeding; and 3) the name, mailing address (if available), and contact information of the party offering or moving the document into evidence. Subsequent to the use of this document in a formal proceeding, it is requested that FISWG be notified as to its use and the outcome of the proceeding. Notifications should be sent to: FISWG@yahoogroups.com.

Redistribution Policy:

FISWG grants permission for redistribution and use of all publicly posted documents created by FISWG, provided that the following conditions are met:

Redistributions of documents, or parts of documents, must retain the FISWG cover page containing the disclaimer.

Neither the name of FISWG, nor the names of its contributors, may be used to endorse or promote products derived from its documents.

Any reference or quote from a FISWG document must include the version number (or creation date) of the document and mention if the document is in a draft status.

Section 3

Guidelines and Recommendations for Facial Comparison Training to Competency

Introduction

With growing use of one-to-one facial examinations and automated facial recognition systems, the need for trained individuals to perform facial comparisons is increasing. In addition, the recommendations provided in the National Academy of Sciences' report, *Strengthening Forensic Science in the United States: A Path Forward*[1], include the need for sufficient training. The purpose of this document is to provide the recommended elements of training to achieve competency in facial comparisons. Future FISWG documents will address the elements described in this document in greater detail. The level of training necessary to conduct facial comparison is dependent upon the quality of images that are being analyzed and the purpose of the analysis.

The task of facial review in an investigative capacity includes, but is not limited to, the use of a facial recognition system to review one-to-many galleries. For example, an officer at a booking station will conduct a one-to-many search of a controlled image against a database of controlled images. This task may also include applications involving high volume throughput. These reviewers require a basic level of training to acquire general knowledge and comprehension of the technology and major elements of the facial comparison discipline.

The task of facial examination includes, but is not limited to, a rigorous one-to-one analysis, comparison, and evaluation of controlled and uncontrolled images for the purpose of effecting a conclusion. Examiners in this situation have to draw on a larger foundation of knowledge, skill, and ability to accurately reach their conclusions. Additionally, the articulation of the scientific and legal basis for the expression of conclusions for many forensic, intelligence, or law enforcement purposes requires an even more advanced level of training to include an expanded set of knowledge, skills, and abilities above the level of basic concepts.

The purpose of this document is to provide guidance on the relevant subject matter to the individual so that upon the completion of training they will be able to conduct comparisons at the basic level or at the advanced level.

Training for Facial Review

Basic Level

General Knowledge:

The trainee should be familiar with the history of facial comparisons in forensic science to include past methods, such as the Bertillion method, and their shortcomings. In addition, they should also understand the perception of facial recognition in the legal community.

Both the principles of individuality and the principles of permanence should be examined. The trainee must be able to distinguish between class and individual characteristics, as well as transient and stable characteristics.

The trainee must understand common terminology and the definitions used within the relevant community, such as the distinction between human facial recognition, automated facial recognition, and facial identification.

An understanding of the principles of comparison should be demonstrated. These principles include:

- Assessment of facial image quality to determine the value for examination

[1] *Strengthening Forensic Science in the United States: A Path Forward* (http://www.nap.edu/catalog.php?record_id=12589#toc)

This document includes a cover page with the FISWG disclaimer

- Process of Analysis, Comparison, Evaluation and Verification (ACE-V)
- Methods of comparisons (see One-to-One Facial Examination Overview)
- Levels of conclusion
- Ability to render proper conclusions
- Overview and effects of cognitive bias, to include confirmation bias
- Understanding of the benefits of verification by a second qualified reviewer/examiner

The trainee should have a general knowledge of automated biometric systems. This includes, but is not limited to, user input and operation, system operation and output, and the limitations of the technology, such as the ability of the technology to distinguish between twins and the impact of disguises and image quality.

The basics of image science are a critical component for the trainee to demonstrate an understanding of, to include:

- Vision
  - Color
  - Illumination
- Photography
  - General principles
  - Hardware and settings
- Perspective
  - Camera to subject distance
  - Angle of view
- Digital images and compression

When applicable, the trainee should be skilled in proper handling of media, write protection of that media, and generating working copies.

Facial Knowledge:

The trainee should be familiar with the bones that comprise the skull and the overlying musculature. An awareness of the varying features of the skin, hair, and hairlines, and their level of permanence, should be obtained. Additionally, anthropometric landmarks, general nomenclature of the facial shapes, and the properties of the ear should be reviewed.

Due to the variable nature of the human face over time, the results of aging must be understood. The trainee should also be aware of other alterations of the face, both temporary and permanent. Examples of temporary changes are: cosmetics, weight changes, hair color changes, wounds, and abrasions. Permanent changes may include: scars, surgical alterations, dental changes, tattoos, and piercings.

Legal/Justice Issues (for examiners who may testify):

Individuals testifying to facial review must be aware of the implications of the relevant judicial decisions that govern admittance of scientific evidence in court. Additionally, attention must be paid to issues such as proper chain of custody, documentation and notes, reporting of results, and technical review.

The trainee should be aware of common misconceptions created by popular media to include fictional television shows, novels, and movies, cumulatively known as 'The CSI Effect'.

Training for Facial Examination

Advanced level

General Knowledge:

The trainee must be able to summarize the history of facial comparisons in forensic science to include past methods, such as the Bertillion method, and their shortcomings. In addition, they should be able to demonstrate knowledge of the perception of facial recognition in the legal community. Likewise, the trainee must be able to summarize the history of forensic photographic comparisons.

The trainee must be able to define the principles of individuality and the principles of permanence. The differences between class and individual, as well as transient and stable characteristics, must also be compared and contrasted.

This document includes a cover page with the FISWG disclaimer

The trainee must be able to apply common terminology and the definitions used within the relevant community, to include the distinction between human facial recognition, automated facial recognition, and facial identification.

A comprehensive working knowledge of the principles of comparison must be demonstrated. These principles include:
- Assessment of facial image quality to determine the value for examination
- Process of Analysis, Comparison, Evaluation and Verification (ACE-V)
- Methods of comparisons (see One-to-One Facial Examination Overview)
- Models of Evaluation and Interpretation
- Levels of conclusion
- Ability to render proper conclusions
- Overview and effects of cognitive bias, to include confirmation bias
- Understanding of the benefits and limitations of review by a second qualified reviewer/examiner

The trainee must have an in-depth knowledge of automated biometric systems. If the agency does not utilize an automated biometrics system, the trainee should have a general knowledge of these systems. This knowledge includes user input and operation, system operation and output, and the factors that affect the performance of the technology, such as the ability to distinguish between twins and the impact of disguises and image quality. Additionally, general biometric matching algorithms should be understood.

The trainee must demonstrate and understand the principles of image science to assist in predicting the effects of photographic processes. This includes:
- Vision
  - Color
  - Illumination
  - Perception
- Photography
  - General principles and theory
  - Hardware and settings
  - Lens properties and potential distortions
  - Illumination of scene and subject
  - Human factors, such as pose and expression
- Perspective
  - Camera to subject distance
  - Angle of view
- Components of digital images and compression
  - Knowledge of sensors, pixels, and resolution
- Methods for the detection of manipulation within images
- Properties of video

The trainee must be skilled in proper handling of media, write protection of that media, and generation of working copies.

Tasks involving image processing may be necessary in facial examination. Therefore, the trainee must demonstrate competency in performance of a range of processing tasks to include, but not limited to, the following:
- Brightness and contrast adjustments
- Rotations and cropping
- Sharpening and blurring
- Scaling and overlays
- Color channel separation
- Effects of image adjustments and enhancements

Facial Knowledge:

The trainee must be able to locate the bones that comprise the skull. Similarly, the knowledge of the overlying musculature and anatomical function must be articulated. The trainee must demonstrate a thorough understanding of the varying features of the skin, hair, and hairlines, and their level of permanence. Additionally, general nomenclature of the facial shapes must be demonstrated. The trainee should be aware of available and relevant statistics regarding facial shapes and relative frequency of occurrence within the general population and subpopulations (e.g., ethnic groups).

Knowledge of the history of ear comparisons, to include the distinction between analysis of ear prints and ear imagery,

This document includes a cover page with the FISWG disclaimer

must be described. An ability to identify the features of the ear and knowledge of the general nomenclature must be demonstrated.

Due to the variable nature of the human face over time, the results of aging must be understood to include, but not be limited to, predicting the common locations of wrinkles. The trainee must also be aware of other alterations of the face, both temporary and permanent. Examples of temporary changes are: cosmetics, weight changes, hair color changes, wounds, and abrasions. Permanent changes may include: scars, surgical alterations, dental changes, tattoos, and piercings.

Legal/Justice Issues (for examiners who may testify):

Individuals testifying to facial examinations must know the implications of the relevant judicial decisions that govern admittance of scientific evidence in court. Additionally, attention must be paid to issues such as proper chain of custody, documentation and notes, reporting of results, and technical review. The history of photographic comparisons in court and relevant case law should be understood.

The trainee must be competent in explaining the process of facial examinations to the jury, the limits of the relevant science and technology, and the creation of visual aids. This competency should be demonstrated through the process of moot courts and/or mock trials.

Lastly, the trainee should be aware of common misconceptions created by popular media to include fictional television shows, novels, and movies, cumulatively known as 'The CSI Effect'.

This document includes a cover page with the FISWG disclaimer

Reference List

FISWG documents can be found at:
www.FISWG.org

Section	Title
Section 1	Glossary
Section 2	Facial Comparison Overview
Section 3	Guidelines and Recommendations for Facial Comparison Training to Competency
Section 4	Guidelines for Specifications, Procurement, Deployment, and Operations of Facial Recognition Systems
Section 5	
Section 6	
Section 7	
Section 8	
Section 9	
Section 10	

This document includes a cover page with the FISWG disclaimer
