# Supplementary material for: Do professional facial image comparison training courses work?
Source: PLoS One. 2019 Feb 13;14(2):e0211037. doi: 10.1371/journal.pone.0211037 (PMC6373902; doi:10.1371/journal.pone.0211037)
Supplement: S3 Appendix — (DOCX) [file pone.0211037.s003.docx]

**S3 Appendix. Supplementary analyses and task descriptions**

**Online Training Course Evaluation Analyses**

Accuracy data for the GFMT, GBU-unlimited and GBU-4 seconds were analysed separately using three 3 x 2 ANOVAs with Training (Course A, Course B, control) as a between-subjects factor and Test (pre-training and post-training) as a within-subjects factor.

*The Glasgow Face Matching Test*

The main effects of Training [*F*<1, *ƞ_p_^2^* = .01] and Test [*F*(1, 87) = 1.98, *p* > .05, *ƞ_p_^2^* = .02], and the interaction between Training and Test [*F*<1, *ƞ_p_^2^* = .01] were non-significant. Therefore, accuracy on the GFMT did not improve following any of the training courses. The average accuracy observed on the GFMT (*M* = 81.4%, *SD* = 11.2%) is consistent with normative data on this test (*M* = 81.3%, *SD* = 9.7%; Burton, White, & McNeill, 2010).

*GBU Tests*

For the GBU-unlimited, the main effects of Training [*F*<1, *ƞ_p_^2^* = .01] and Test [*F*<1, *ƞ_p_^2^* = .00], and the interaction between Training and Test [*F*<1, *ƞ_p_^2^* = .00] were non-significant. For the GBU-4 seconds, the main effects of Training [*F*<1, *ƞ_p_^2^* = .02] and Test [*F*(1, 87) = 1.31, *p* > .05, *ƞ_p_^2^* = .02], and the interaction between Training and Test [*F*<1, *ƞ_p_^2^* = .02] were also non-significant. Therefore, accuracy on the GBU tests did not improve following any of the training courses.

**Half-Day Face-to-Face Training Course Evaluation Analyses**

*Glasgow Face Matching Test*

GFMT accuracy data were analysed using a 2 x 2 ANOVA with Training (Course C, control) as a between-subjects factor and Test (pre-training, post-training) as a within-subjects factor. Overall, Course C trainees (88%) outperformed control participants (78%), as indicated by a significant main effect of Training [*F*(1, 244) = 50.01, *p* < .001, *ƞ_p_^2^* = .17]. Importantly however, the main effect of Test [*F*(1, 244) = 2.54, *p* > .05, *ƞ_p_^2^* = .01] and the interaction between Training and Test were non-significant [*F*(1, 244) = 1.48, *p* > .05, *ƞ_p_^2^* = .01], indicating these group differences were not a result of training.

*High-to-Low image quality test*

Accuracy data on the High-to-Low image quality test were analysed using a 2 x 2 ANOVA with Training (Course C, control) as a between-subjects factor and Test (pre-training, post-training) as a within-subjects factor. Course C trainees (83%) outperformed control participants (80%), as indicated by a significant main effect of Training [*F*(1, 244) = 4.43, *p* < .05, *ƞ_p_^2^* = .02]. However, the main effect of Test [*F*<1, *ƞ_p_^2^* = .00] and the interaction between Training and Test were non-significant [*F*<1, *ƞ_p_^2^* = .00], indicating no improvement from training.

*High-to-High image quality test*

Accuracy data on the High-to-High image quality test were analysed using a 2 x 2 ANOVA with Training (Course C, control) as a between-subjects factor and Test (pre-training, post-training) as a within-subjects factor. Consistent with the previous tests, Course C trainees (98%) outperformed control participants (96%) [main effect of Training: *F*(1, 244) = 9.54, *p* < .05, *ƞ_p_^2^* = .04], but the main effect of Test [*F*<1, *ƞ_p_^2^* = .00] and the interaction between Training and Test were non-significant [*F*<1, *ƞ_p_^2^* = .00], indicating no improvement from training. Accuracy on this test was at ceiling and so it is not surprising that we did not observe a training effect here.

*Effect of training on untrained participants*

More than half of the Course C trainees reported having previously received training in facial image comparison. To determine if previous training diluted the interaction effect we repeated the analyses above including only those who had not received prior training (*n* = 99). For the GFMT, the main effect of Training [*F*(1,139) = 33.01, *p* < .001, *ƞ_p_^2^* = .19] was significant, but the main effect of Test [*F*(1, 139) = 2.78, *p* > .05, *ƞ_p_^2^* = .02] and the interaction between Training and Test [*F*<1, *ƞ_p_^2^* = .00] were non-significant. For the High-to-Low test, the main effects of Training [*F*(1, 139) = 3.35, *p* > .05, *ƞ_p_^2^* = .02] and Test [*F*<1, *ƞ_p_^2^* = .00], and the interaction between Training and Test [*F*(1, 139) = 2.17, *p* > .05, *ƞ_p_^2^* = .02] were non-significant. For the High-to-High test, the main effect of Training [*F*(1, 139) = 5.98, *p* < .05, *ƞ_p_^2^* = .04] was significant, but the main effect of Test [*F*<1, *ƞ_p_^2^* = .00] and the interaction between Training and Test [*F*<1, *ƞ_p_^2^* = .00] were non-significant. These analyses are consistent with the reported pattern of results. A t-test also revealed no differences in overall accuracy (collapsing across the three identification tasks) between those previously trained and untrained at both pre-training [*t*(202) = .19, *p* > .05] and post-training [*t*(202) = 1.28, *p* > .05]. These analyses confirm that Course C did not improve face identification accuracy.

**3-Day Face-to-Face Training Course Evaluation**

*Tests*

*Inversion Test.* The inversion effect is a measure of visual expertise, with experts in a particular domain usually demonstrating a stronger inversion effect (i.e., a larger impairment). However, this standard expertise explanation for the inversion effect may not hold for experts in facial image comparison, as they tend to show a *reduced* inversion effect compared to controls ([Towler, White, & Kemp, 2017](#_ENREF_8); [White, Phillips, Hahn, Hill, & O'Toole, 2015](#_ENREF_10)). We therefore wanted to track any changes to this effect from before to after training.

During training, Course D trainees made same/different identity decisions on a set of male model images that were not included in the pre- or post-training inversion tests. Afterwards, for each comparison, the trainer took a poll of trainees’ answers and revealed the correct answer. The trainer then facilitated a lengthy group discussion about why each face pair was the same person or different people. This discussion focussed on identifying diagnostic facial features and their sub-parts, and encouraged trainees to compare the faces piece by piece instead of relying on holistic whole-face judgements. The group did not move on to the next comparison until everyone in the group was satisfied they could see why the images showed the same person or different people.

*CFMT.* The Cambridge Face Memory Test ([Duchaine & Nakayama, 2006](#_ENREF_2)) and the more recent Cambridge Face Memory Test – Australian ([McKone et al., 2011](#_ENREF_5)) are standardised tests of novel face learning. Using itemised performance data for both tests ([see McKone et al., 2011](#_ENREF_5)) we created two equally difficult versions of what we will refer to as the Cambridge Face Memory Test – modified (CFMT-m), where items from both original tests were combined in equal proportions. As in the two original tests, participants learn six identities over 18 trials, and then complete a 54-item recognition test under increasingly difficult conditions at both pre- and post-training. Test version order was randomly allocated to pre- and post-training for each participant. We included the CFMT-m to check that any training improvement was not simply a face-specific improvement.

*Embedded Figures Test.* The Embedded Figures Test measures field dependence ([see Witkin, Oltman, Raskin, & Karp, 1971](#_ENREF_11)), whereby those who are field-independent find it easier to ignore the background in order to find a target, and those that are field-dependent find it much harder to ignore the background. Given evidence of enhanced feature-based processing in facial examiners ([see Towler et al., 2017](#_ENREF_8)), we predicted that performance on the embedded figures test might correlate positively with performance on the face matching tasks, and may be stronger at post-training for the Course D trainees (see Table S1 and S2). We downloaded 54 embedded figures stimuli ([see IndiaBix Technologies, 2017](#_ENREF_3)), each of which featured a target pattern which participants were to find in one of four complex line drawings. Using pilot item accuracy data, we selected 38 of the most difficult trials and split them into two equally difficult versions of 19 items each. Allocation of each version to the pre- and post-training was randomised for each participant.

*Matching Familiar Figures.* The Matching Familiar Figures Test (MFFT) is a test of the cognitive style of impulsivity versus reflexivity ([Kagan, 1965](#_ENREF_4)), and correlates positively with unfamiliar face matching accuracy ([Burton et al., 2010](#_ENREF_1); [Megreya & Burton, 2006](#_ENREF_6)). We predicted this effect may be stronger at post-training for the Course D trainees (see Table S1 and S2). Participants were presented with a line drawing of a target object (e.g., a tree) and asked to find the identical object amongst an array of six other similar drawings. Using pilot item accuracy data we created two equally difficult versions of the test, each containing 24 items. Allocation of each version to the pre- and post-training was randomised for each participant.

*Navon Task.* The Navon Task ([Navon, 1977](#_ENREF_7)) is a measure of global precedence, whereby participants are presented with a big ‘global’ letter (e.g., E) made up of a small ‘local’ letter (e.g., m), and are usually faster to identify the big letter than the small letter. In this version of the task, participants completed 10 blocks of 10 trials at pre- and post-training, where in each block they reported whether a randomly selected target letter was the big or small letter in each of the trial stimuli. Given enhanced feature-based processing in facial examiners ([see Towler et al., 2017](#_ENREF_8)), we predicted that reaction time on the small letter trials might correlate negatively with performance on the face matching tasks, and that it would be strongest at post-training for the Course D trainees (see Table S1 and S2).

*Full Analyses*

*Glasgow Face Matching Test.* We analysed GFMT accuracy data using a 2 x 2 mixed ANOVA with Training (Course D *n* = 29, controls *n* = 20) as a between-subjects factor and Test (pre-test, post-test) as a within-subjects factor. The main effects of Training [*F*(1, 47) = 1.27, *p* > .05, *ƞ_p_*^2^ = .03] and Test [*F*<1, *ƞ_p_*^2^ = .01] were non-significant. However, the interaction between Training and Test was significant [*F*(1, 47) = 4.69, *p* < .05, *ƞ_p_*^2^ = .09]. Simple main effects analyses confirmed a significant benefit of training for the Course D trainees [*F*(1, 47) = 5.47, *p* < .05] but not for the control group [*F*<1]. The 6% improvement in the Course D trainees is equivalent to making one additional correct decision at post-test than pre-test.

*Casework Test.* We analysed accuracy on the casework test using a 2 x 2 mixed ANOVA with Training (Course D *n* = 30, controls *n* = 20) as a between-subjects factor and Test (pre-training, post-training) as a within-subjects factor. The main effects of Training [*F*<1, *ƞ_p_*^2^ = .01] and Test [*F*<1, *ƞ_p_*^2^ = .00] were non-significant. Importantly, the interaction between Training and Test was also non-significant [*F*<1, *ƞ_p_*^2^ = .02].

*Feature Rating Task.* We calculated the extent to which participants’ feature similarity ratings were diagnostic of identity. To do this, we computed AUC for each participant using their average feature similarity ratings for each item as the predictor variable. We analysed this AUC data using a 2 x 2 mixed ANOVA with Training (Course D *n* = 28, controls *n* = 20) as a between-subjects factor, and Test (pre-training, post-training) as a within-subjects factor. The main effect of Test [*F*(1, 46) = 8.43, *p* < .05, *ƞ_p_*^2^ = .16] was significant. The main effect of Training [*F*(1, 46) = 2.16, *p* > .05, *ƞ_p_*^2^ = .05], and the interaction between Training and Test [*F*<1, *ƞ_p_*^2^ = .00] were non-significant.

We also analysed accuracy data on the feature rating task using a 2 x 2 x 2 mixed ANOVA with Training (Course D *n* = 28, controls *n* = 20) as a between-subjects factor, and Test (pre-training, post-training) and Ratings (no ratings, ratings) as within-subjects factors. The main effect of Training was non-significant [*F*(1, 46) = 2.76, *p* > .05, *ƞ_p_*^2^ = .06], indicating no overall difference in accuracy between the Course D trainees and control participants. The main effect of Test was significant [*F*(1, 46) = 12.58, *p* < .05, *ƞ_p_*^2^ = .22], indicating both groups improved from pre- to post-training. The main effect of Ratings was also significant [*F*(1, 46) = 10.73, *p* < .05, *ƞ_p_*^2^ = .19], indicating increased accuracy when participants rated the similarity of facial features prior to making a same/different identity decision, consistent with previous research ([see Towler et al., 2017](#_ENREF_8)). The two-way interactions between Training and Test [*F*(1, 46) = 2.61, *p* > .05, *ƞ_p_*^2^ = .05], Training and Ratings [*F*<1, *ƞ_p_*^2^ = .01], and Test and Ratings [*F*(1, 46) = 2.52, *p* > .05, *ƞ_p_*^2^ = .05] were non-significant, as was the three-way interaction between Training, Test and Ratings [*F*<1, *ƞ_p_*^2^ = .01].

*Inversion Test.* We analysed accuracy data on the inversion test using a 2 x 2 x 2 mixed ANOVA with Training (Course D *n* = 30, controls *n* = 20) as a between-subjects factor, and Test (pre-training, post-training) and Orientation (upright, inverted) as within-subjects factors. The main effect of Training [*F*<1, *ƞ_p_*^2^ = .00] was non-significant, but there was a significant main effect of Test [*F*(1, 48) = 14.65, *p* <.001, *ƞ_p_*^2^ = .23] and Orientation [*F*(1, 48) = 76.69, *p* < .001, *ƞ_p_*^2^ = .62]. The interaction between Training and Test [*F*(1, 48) = 4.11, *p* < .05, *ƞ_p_*^2^ = .08] was significant, but the interactions between Training and Orientation [*F*<1, *ƞ_p_*^2^ = .02] and Test and Orientation [*F*(1, 48) = 3.12, *p* > .05, *ƞ_p_*^2^ = .06] were non-significant. The three-way interaction between Orientation, Training and Test was non-significant [*F*<1, *ƞ_p_*^2^ = .00], indicating equivalent inversion effects for Course D trainees and control participants from pre- to post-training. To follow-up the significant interaction between Training and Test we conducted simple main effects analyses. These revealed a significant improvement pre- to post-training for Course D trainees [*F*(1, 48) = 21.42, *p* < .001] but not the control participants [*F*(1, 48) = 1.35, *p* > .05].

*Cambridge Face Memory Test-modified*. We analysed the CFMT-m accuracy data using a 2 x 2 mixed ANOVA with Training (Course D *n* = 31, controls *n* = 20) as a between-subjects factor and Test (pre-training, post-training) as a within-subjects factor. The main effect of Training [*F*<1, *ƞ_p_*^2^ = .00] and the interaction between Training and Test were non-significant [*F*<1, *ƞ_p_*^2^ = .00]. The main effect of Test [*F*(1, 49) = 26.61, *p* < .001, *ƞ_p_*^2^ = .35] was significant, indicating that both groups performed significantly worse on the CFMT-m after training.

*Individual Differences*

|  | Casework | | Feature Ratings Task | | | | Inversion Test | | | | GFMT | |
| --- | --- | --- | --- | --- | --- | --- | --- | --- | --- | --- | --- | --- |
|  |  |  | No Ratings | | Ratings | | Upright | | Inverted | |  |  |
|  | Pre | Post | Pre | Post | Pre | Post | Pre | Post | Pre | Post | Pre | Post |
| Embedded Figures Test | | | | | | | | | | | | |
| Pre-training | -.033 | .309 | .461* | .231 | .369 | .350 | .371* | -.179 | -.034 | .183 | .168 | .078 |
| Post-training | .056 | .421* | .658** | .293 | .265 | .372 | .173 | -.045 | .031 | .087 | .164 | -.019 |
| MFFT | | | | | | | | | | | | |
| Pre-training | .285 | .385* | .476* | .008 | .205 | .067 | .034 | .233 | .098 | .055 | .158 | .100 |
| Post-training | .075 | .070 | .168 | .137 | .171 | .102 | -.228 | .232 | .011 | -.058 | .310 | .312 |
| Navon Task (small letter RT) | | | | | | | | | | | | |
| Pre-training | -.413* | -.156 | -.022 | -.199 | .168 | -.252 | -.076 | .194 | -.066 | .183 | .221 | .226 |
| Post-training | -.223 | .156 | .082 | -.158 | .231 | -.064 | -.096 | .099 | .029 | .202 | .375* | .244 |

**Table S1.** *Correlations between individual difference measures (embedded figures test, MFFT and the Navon task) and face matching tasks for Course D trainees participants. * significant at the .05 level, ** significant at the .01 level.*

|  | Casework | | Feature Ratings Task | | | | Inversion Test | | | | GFMT | |
| --- | --- | --- | --- | --- | --- | --- | --- | --- | --- | --- | --- | --- |
|  |  |  | No Ratings | | Ratings | | Upright | | Inverted | |  |  |
|  | Pre | Post | Pre | Post | Pre | Post | Pre | Post | Pre | Post | Pre | Post |
| Embedded Figures Test | | | | | | | | | | | | |
| Pre-training | -.162 | .016 | .523* | .294 | .072 | -.035 | -.030 | .336 | .267 | .449* | -.228 | .100 |
| Post-training | -.074 | -.060 | .476* | .079 | .213 | -.059 | .044 | .247 | .155 | .539* | -.052 | .078 |
| MFFT | | | | | | | | | | | | |
| Pre-training | -.275 | -.461* | .351 | -.090 | -.181 | .331 | -.066 | .032 | .431 | -.265 | -.143 | .074 |
| Post-training | .041 | -.567** | .287 | -.054 | -.148 | .055 | -.146 | -.037 | .456* | .023 | -.050 | .106 |
| Navon Task (small letter RT) | | | | | | | | | | | | |
| Pre-training | .524* | -.266 | -.054 | -.158 | .032 | -.153 | -.136 | -.058 | .245 | .757 | .198 | .109 |
| Post-training | .256 | -.015 | .089 | .083 | .015 | -.168 | -.041 | .057 | .208 | .169 | -.040 | -.004 |

**Table S2.** *Correlations between individual difference measures (embedded figures test, MFFT and the Navon task) and face matching tasks for control participants. * significant at the .05 level, ** significant at the .01 level.*

**Bayesian Analyses**

Across the three experiments we found a number of non-significant interactions between Training and Test. In these cases, we assessed the evidence for the null hypothesis, i.e. that training does not improve accuracy, by conducting Bayesian repeated measures ANOVAs with a between-subjects factor of Training (training group/s, control) and a within-subjects factor of Test (pre-training, post-training) using JASP (0.9.0.1). We used the default Cauchy prior (r = .5), but verified that our results were robust to prior specification. We looked at the likelihood of inclusion of each effect focussing on the interaction between Training and Test ([see Wagenmakers, 2007](#_ENREF_9)). The Bayes factors reported in Table S3 indicate the number of times more likely the observed data is to occur in models that do not include an interaction between Training and Test (evidence for no effect of training) versus models that do include an interaction between Training and Test.

|  |  | Evidence for no effect of training | | | | |
| --- | --- | --- | --- | --- | --- | --- |
| Course | | Extreme | Very Strong | Strong | Moderate | Anecdotal |
| A & B | *GFMT* |  | 33 |  |  |  |
|  | *GBU-u* | 111 |  |  |  |  |
|  | *GBU-4s* |  | 31 |  |  |  |
| C | *GFMT* |  |  |  | 5 |  |
|  | *High-to-Low* |  |  | 11 |  |  |
|  | *High-to-High* |  | 14 |  |  |  |
| C *untrained* | *GFMT* |  |  |  | 4 |  |
|  | *High-to-Low* |  |  |  | 4 |  |
|  | *High-to-High* |  |  | 13 |  |  |
| D | *Casework test* |  |  | 12 |  |  |

**Table S3.** *Bayes factors across all three experiments indicating the number of times more likely the observed data are to occur in models that do not include an interaction between Training and Test and thus provide evidence for no effect of training, versus models that do include an interaction.*

**References**

Burton, A. M., White, D., & McNeill, A. (2010). The Glasgow Face Matching Test. *Behavior Research Methods, 42*(1), 286-291. doi:10.3758/BRM.42.1.286

Duchaine, B. C., & Nakayama, K. (2006). The Cambridge Face Memory Test: Results for neurologically intact individuals and an investigation of its validity using inverted face stimuli and prosopagnosic participants. *Neuropsychologia, 44*, 576-585. doi:10.1016/j.neuropsychologia.2005.07.001

IndiaBix Technologies. (2017). Non verbal reasoning: Embedded images. Retrieved from <www.indiabix.com/non-verbal-reasoning/embedded-images/>

Kagan, J. (1965). Reflection-impulsivity and reading ability in primary grade children. *Child Development, 36*, 609-628.

McKone, E., Hall, A., Pidcock, M., Palermo, R., Wilkinson, R. B., Rivolta, D., . . . O'Connor, K. B. (2011). Face ethnicity and measurement reliability affect face recognition performance in developmental prosopagnosia: Evidence from the Cambridge Face Memory Test–Australian. *Cognitive Neuropsychology, 28*(2), 109-146.

Megreya, A. M., & Burton, A. M. (2006). Unfamiliar faces are not faces: Evidence from a matching task. *Memory and Cognition, 34*(4), 865-876.

Navon, D. (1977). Forest before trees - Precedence of global features in visual-perception. *Cognitive Psychology, 9*, 353-383.

Towler, A., White, D., & Kemp, R. I. (2017). Evaluating the feature comparison strategy for forensic face identification. *Journal of Experimental Psychology: Applied, 23*(1), 47-58. doi:<http://dx.doi.org/10.1037/xap0000108>

Wagenmakers, E. J. (2007). A practical solution to the pervasive problems of p values. *Psychon Bull Rev, 14*(5), 779-804.

White, D., Phillips, P. J., Hahn, C. A., Hill, M., & O'Toole, A. J. (2015). Perceptual expertise in forensic facial image comparison. *Proceedings of the Royal Society of London B: Biological Sciences, 282*, 1814-1822.

Witkin, H. A., Oltman, P. K., Raskin, E., & Karp, S. (1971). *A manual for the embedded figures test.* California: Consulting Psychologists Press.
